# Nonclonal *Burkholderia pseudomallei* Population in Melioidosis Case Cluster, Sri Lanka

**DOI:** 10.3201/eid2711.210219

**Published:** 2021-11

**Authors:** Himali S. Jayasinghearachchi, Vaithehi R. Francis, Harindra D. Sathkumara, Shivankari Krishnananthasivam, Jayanthi Masakorala, Thilini Muthugama, Aruna D. De Silva, Enoka M. Corea

**Affiliations:** General Sir John Kotelawala Defense University, Ratmalana, Sri Lanka (H.S. Jayasinghearachchi, T. Muthugama, A.D. De Silva); Eastern University, Vantharumoolai, Sri Lanka (V.R. Francis); Genetech Research Institute, Colombo, Sri Lanka (H.D. Sathkumara, S. Krishnananthasivam); University of Colombo, Colombo (J. Masakorala, E.M. Corea)

**Keywords:** *Burkholderia pseudomallei*, melioidosis, sequence type, nonclonal, case cluster, bacteria, Sri Lanka

## Abstract

A melioidosis case cluster of 10 blood culture–positive patients occurred in eastern Sri Lanka after an extreme weather event. Four infections were caused by *Burkholderia pseudomallei* isolates of sequence type 594. Whole-genome analysis showed that the isolates were genetically diverse and the case cluster was nonclonal.

Melioidosis, an emerging tropical infection caused by the soil bacterium *Burkholderia pseudomallei*, is found most commonly in northern Australia and the tropical countries of Southeast Asia. Melioidosis is endemic in Sri Lanka and has a case-fatality rate of 24% (*1*). The primary route of acquisition is inoculation of contaminated surface water or soil through skin and mucous membranes. However, a high incidence of pneumonia with sepsis has been reported during extreme weather events, such as heavy rainfall, indicating that inhalation of aerosolized bacteria during cyclones and typhoons is a likely mode of transmission in this setting (*2*).

We identified a case cluster of 10 blood culture–positive cases of melioidosis in the Batticaloa District of the Eastern Province of Sri Lanka during November–December 2015, after a flooding event; 4 case-patients died (Appendix Table). Before this case cluster was identified, 1 case had been reported from Batticaloa in March 2015, and after this case cluster, 5 cases were found in 2016. We confirmed isolates as *B. pseudomallei* by real-time PCR to detect the *lpxo* gene (*3*). We identified 6 sequence types (STs) by multilocus sequence typing (MLST) (*4*); of the 10 isolates, 4 were ST594, belonging to the uncommon *B. thailandensis*–like flagellum and chemotaxis (BTFC) gene cluster. Although the monthly average rainfall for October in the Batticaloa District is usually 160 mm, ≈331.5 mm of rain was recorded in the 48 hours ending at 8:30 a.m. Previous evidence has shown that, in this setting, infections are usually caused by diverse strains because of widespread aerosolization of multiple clones (*2*). We performed whole-genome sequencing (WGS) to determine clonality among the isolates of identical ST. The Ethics Review Committee of the Faculty of Medicine, University of Colombo, (Colombo, Sri Lanka), approved this study.

We mapped the geographic location of the cases on Google Earth using ArcGIS version 10.1 (ESRI, https://www.esri.com) (Figure). We compared WGS data of 3 ST594 *B. pseudomallei* isolates (114, 122, and 133) using binary alignment files with Unipro UGENE version 33 (*5*). We determined the distribution of genomic islands (GIs) using the Island Viewer 4 web tool (*6*) and the National Center for Biotechnology Information Multiple Sequence Alignment (https://www.ncbi.nlm.nih.gov/tools/msaviewer) and BLAST (https://blast.ncbi.nlm.nih.gov/Blast.cgi) tools. We used the PHAge Search Tool Enhanced Release (PHASTER) webserver to analyze the annotated bacterial genomes to locate prophage-specifying DNA regions (*7*). We manually searched the sequences identified by PHASTER and used the position of integrase and last phage–related genes to determine genome boundaries (*8*). We predicted and annotated all open reading frames (ORFs) of the prophages by PHASTER and BLASTp. We also determined the intracellular motility factor (*bimA*) allele type present in each strain as previously described (*9*). 

**Figure:**
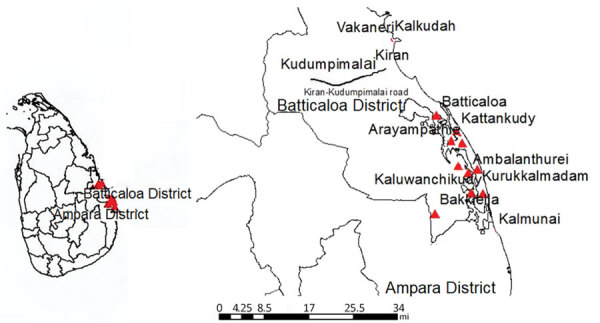
Location of melioidosis cases (red arrows) in the Batticaloa District of Sri Lanka during November–December 2015. Inset shows location of Batticaloa and Ampara Districts in Sri Lanka.

WGS showed genetic diversity in terms of gene content, location, number and type of GIs, prophage DNA distribution, number of intact prophages and *bimA* allele type among the isolates of ST594 (Table). These results show that the case cluster was nonclonal in origin.

**Table:** Characteristics of *Burkholderia pseudomallei* isolates from cluster cases of melioidosis after heavy rain, Sri Lanka*

Characteristic	Isolate 114	Isolate 122	Isolate133
Sequence type	594	594	594
Clade	BTFC	BTFC	BTFC
*BimA* allele type	*BimA*_Bm_	*BimA*_Bp_	*BimA*_BP_
No. prophages in chromosome 1	6	1	6
No. prophages in chromosome 2	1	1	1
No. intact phages	0	2	0
Presence or absence of intact prophage PHAGE_Burkho_phiE202_NC_009234	Absent	Present	Absent
GIs identified	GI1, GI4, GI5, GI6, GI7, GI8, GI9, GI8.i, GI8.ii, GI11, GI12, GI13, GI14, GI16	GI1. GI2, GI4, GI6, GI7, GI8, GI8.i, GI8.ii, GI11, GI12, GI13, GI14, GI16	GI1, GI4, GI6, GI7, GI8, GI8.i, GI8.ii, GI11, GI12, GI13, GI14, GI16
GIs located next to tRNA genes	GI7	GI2, GI4	GI7, GI8
GIs containing phage or site-specific integrases	GI4, GI7, GI15i	GI7, GI15i	GI4, GI7, GI15i
GIs containing prophage DNA	GI7, GI4, GI15i, GI13	GI2, GI7, GI15i, GI13	GI4, GI7, GI15i, GI13
No. transposable elements in GI8	7	6	6
New GI15i	GI15i	GI15i	GI15i
No. integrases	2	2	2

*BTFC, *B. thailandensis*–like flagellum and chemotaxis; GI, genomic island.

This case cluster after heavy rainfall and flooding in Sri Lanka confirmed that *B. pseudomallei* is present in the environment of eastern Sri Lanka. The case-fatality rate of the cluster (40%) was almost double that of sporadic infections in Sri Lanka (23%) (*1*). Six of the 10 isolates in our study belonged to diverse STs; 4 isolates were novel (ST1364, ST1442, ST1179, and ST1413). However, 4 isolates belonging to ST594 were from patients from different geographic locations. In addition, WGS analysis of prophage DNA distribution was able to discriminate between the isolates. GI variation in *B. pseudomallei* is known to be associated with genomic plasticity of the organism, and GIs appear to be a main source of genomic diversity within *B. pseudomallei* that can be useful in identifying genetically diverse strains (*10*). The strain-specific variations in the structure and distribution of GIs in the isolates in this case cluster indicate the presence of genetically diverse strains. 

As weather patterns change globally and include more severe weather events and increased flooding, we may see more such case clusters of melioidosis. Physicians in tropical regions must be vigilant for such occurrences because of the high mortality rate associated with melioidosis in this setting. Primary prevention of melioidosis is difficult because of the saprophytic nature of *B. pseudomallei* and regular flooding during the rainy season. The predominance of diabetic patients in this cluster (Appendix Table) illustrates the importance of detection and control of diabetes in high-risk communities for primary prevention. Secondary prevention requires close coordination between clinicians and microbiologists to encourage patients to seek medical advice early in illness and to transfer febrile patients promptly to centers with blood culture facilities. Early suspicion of melioidosis will enable timely, effective antimicrobial treatment.

**Appendix.** Additional information about a nonclonal *Burkholderia pseudomallei* population in melioidosis case cluster, Sri Lanka.
