# Supplementary material for: Nonclonal Burkholderia pseudomallei Population in Melioidosis Case Cluster, Sri Lanka
Source: Emerg Infect Dis. 2021 Nov;27(11):2955–7. doi: 10.3201/eid2711.210219 (PMC8545001; doi:10.3201/eid2711.210219)
Supplement: Supplementary file 1 — Appendix. Additional information about a nonclonal Burkholderia pseudomallei population in melioidosis case cluster, Sri Lanka. [file 21-0219-Techapp-s1.pdf]

Article DOI: https://doi.org/10.3201/eid2711.210219

Nonclonal *Burkholderia pseudomallei* Population in Melioidosis Case Cluster, Sri Lanka

Appendix

Appendix Table. Characteristics of melioidosis case-patients, eastern Sri Lanka*

Isolate	ST (clade)	Age	Sex	Clinical presentation	Underlying conditions	Area	Outcome
BPs110	1152 (BTFC)	29	M	Subcutaneous abscess over chest	None	Kiran	Recovered
BPs109	594 (BTFC)	61	M	Sepsis with pyelonephritis	Diabetes	Kaluwanchikudy	Died
BPs112	1442 (YLF)	66	M	Acute febrile illness (focus of infection not identified)	Diabetes	Kattankudy	Recovered
BPs115	1413 (YLF)	43	F	Pneumonia with pleural effusion	Diabetes	Periyaporathivu	Died
BPs111	1364 (YLF)	33	M	Septic arthritis of elbow	Diabetes	Kiran	Recovered
BPs114	594 (BTFC)	62	F	Sinusitis	Diabetes	Kattankudy	Recovered
BPs116	1179 (BTFC)	20	F	Bronchopneumonia/septic arthritis	None	Kaluwanchikudy	Died
BPs,121	13 (YLF)	18	M	Acute febrile illness (focus of infection not identified)	None	Ambalanthurai	Recovered
BPs122	594 (BTFC)	28	F	Bronchopneumonia/septic arthritis of knee	None	Bakkiella	Died
BPs133	594 (BTFC)	46	M	Sinusitis/thigh abscess	Diabetes	Kattankudy	Recovered

*BPs, *Burkholderia pseudomallei*; BTFC, *B. thailandensis*–like flagellum and chemotaxis; YLF, *Yersinia*-like fimbrial.
